# Non-response to short-term ketamine use for treatment-resistant depression

**DOI:** 10.1007/s43440-025-00730-9

**Published:** 2025-04-30

**Authors:** Michał Walaszek, Wiesław Jerzy Cubała, Zofia Kachlik, Michał Pastuszak, Krzysztof Pastuszak, Aleksander Kwaśny

**Affiliations:** 1 Department of Psychiatry, Faculty of Medicine, Medical University of Gdansk, ul. Smoluchowskiego 17, Gdańsk, 80-214 Poland; 2 Department of Algorithms and System Modeling, Gdansk University of Technology, Gdańsk, Poland; 3 Laboratory of Translational Oncology, Intercollegiate Faculty of Biotechnology, University of Gdańsk and Medical University of Gdańsk, Gdańsk, Poland; 4 Center of Biostatistics and Bioinformatics, Medical University of Gdańsk, Gdańsk, Poland

**Keywords:** Mood disorders, Treatment-resistant depression, Psychopharmacology, Ketamine, Treatment non-response

## Abstract

**Background:**

Ketamine is currently gaining attention as a rapid-acting antidepressant for treatment-resistant depression (TRD). However, many patients fail to respond, and limited data exist on predictors of non-response. This study aims to characterize the sociodemographic and clinical features associated with non-response to ketamine among TRD patients.

**Methods:**

This is a post-hoc analysis of a naturalistic observational study, which enrolled 40 inpatients with treatment-resistant major depressive disorder and analyzed sociodemographic and clinical features in responders and non-responders stratified per Montgomery-Åsberg Depression Rating Scale (MADRS) during short-term ketamine administration (intravenous dosage: 0,5 mg/kg and orally: 2.0 or 2.5 mg/kg) that comprise over 4 weeks.

**Results:**

In this study, 30 patients (75%) were classified as non-responders. No significant differences were detected among sociodemographic and clinical features beyond the history of substance use disorder (SUD) – only 53.3% of non-responders reported prior SUD (vs. 100%; *p* = 0.0075) and a lower number of psychiatric comorbidities (*p* = 0.0381).

**Conclusion:**

This study highlights key characteristics of TRD non-responders to ketamine, including lower rates of SUD and fewer psychiatric comorbidities. These findings suggest that a higher burden of traditional TRD risk factors may not limit ketamine efficacy and could even enhance response compared to “pure” major depressive disorder. Identifying potential non-responders early can optimize treatment decisions, reduce ineffective exposure, and guide future research on improving TRD management.

## Introduction

Major depressive disorder (MDD) is the second leading cause of global years lived with disability (YLDs), accounting for 8.2% of all YLDs worldwide [1]. The lifetime prevalence of MDD is estimated to affect over a quarter of the general population, highlighting its significance as a major public health concern [2]. According to the STAR*D report, one-third of MDD patients may suffer from treatment-resistant depression (TRD) [3]. The regulatory definitions for TRD adopted by the European Medicines Agency (EMA) and the US Food and Drug Administration (FDA) follow consensual TRD definition [4] as the failure to respond to two or more antidepressant regimens, despite adequate dose, duration, and adherence to treatment in the current major depressive episode [5].

The limited efficacy of conventional antidepressants in TRD patients has driven the search for new pharmacological interventions that differ from traditional treatment approaches. These substances, known as rapid-acting antidepressants (RAADs), are characterized by their fast response, with patients often showing improvement within days or even hours. The first RAAD to receive FDA and EMA approval for TRD treatment is an S-ketamine nasal spray, an enantiomer of ketamine and an NMDA receptor (NMDAR) antagonist with an array of other investigative substances demonstrating RAAD in clinical development [5, 6].

Ketamine, a racemic mixture of R-ketamine and S-ketamine, is widely regarded as the prototypical RAAD. While initially believed to exert its effects primarily through glutamatergic modulation, current hypotheses extend beyond this mechanism, implicating the opioid and GABAergic systems, as well as complex secondary messenger pathways. These interactions converge on a cascade of neuroplastic and neurogenic responses, which may underlie its robust clinical effects [7]. Beyond its well-documented antidepressant efficacy, recent findings highlight its rapid anti-suicidal and anti-anhedonic properties, further solidifying its potential as a transformative intervention in psychiatry [8, 9].

This research aims to conduct a post-hoc analysis within a cohort of TRD patients who underwent short-term ketamine intervention in a naturalistic study. This approach seeks to characterize the sociodemographic and clinical features associated with non-response to ketamine.

## Materials and methods

### Participants

This study presents a retrospective cross-sectional analysis of a patient cohort from two naturalistic observational registry protocols examining intravenous (IV) ketamine and oral ketamine interventions in individuals with TRD only in the course of MDD. The methodology used in both registries for treatment-resistant mood disorders is described in detail elsewhere [10, 11]. The study examined individuals exhibiting treatment resistance, defined as at least two adequate and unsuccessful interventions.

Overall, 40 patients were selected from NCT04226963 and NCT05565352. They include 28 patients with IV ketamine intervention and 12 with oral ketamine administration. Both studies received approval from the Independent Bioethics Committee for Scientific Research at the Medical University of Gdańsk, Poland (NKBBN/172–674/2019 and NKBBN/172–447/2022). The research was conducted in compliance with the latest version of the Declaration of Helsinki. All participants provided written informed consent for participation and data collection.

### Study design

The registered studies aimed to evaluate the tolerability and safety profile of eight intravenous (*iv*) ketamine infusions or oral ketamine solutions administered as an adjunctive treatment. The *iv* ketamine was delivered at a dose of 0.5 mg/kg. A detailed description of the *iv* ketamine study design is presented elsewhere [10]. The therapeutic regimen for oral ketamine intervention consisted of eight ketamine administrations over a 4-week duration in conjunction with standard care practices. Oral ketamine solution was administrated at dose of 2.0 or 2.5 mg/kg based on the patient’s actual body weight.

### Outcome measures

Patients were categorized into two groups: *responders* and *non-responders*. A treatment response was defined as a 50% or greater reduction in the *Montgomery-Åsberg Depression Rating Scale* (MADRS) score at the 7th ketamine administration (before the administration and regardless of formulation) compared to the initial score. The 7th administration was chosen to differentiate between responders and non-responders, as it is the final measurement during the acute intervention, with the next assessment occurring at the 7-day follow-up after the last administration.

### Statistical methods

Binary variables were analyzed using Fisher’s exact test. For categorical variables with more than two levels, Fisher’s exact test with the Freeman-Halton extension was applied. The normality of quantitative variables was assessed using the Shapiro-Wilk test. For normally distributed variables, comparisons were made using the Student’s t-test; otherwise, the Mann-Whitney U test was applied. A p-value less than 0.05 was considered statistically significant. No data imputation was performed.

## Results

In this study, 30 patients (75%) were classified as non-responders. Sociodemographic factors such as age, gender, BMI, education level, employment, marital status, household occupancy, and years of education showed no significant differences between responders and non-responders (Table 1). Similarly, baseline clinical data did not reveal any significant distinctions between groups (Table 2). However, two notable differences emerged: the history of substance use disorder (SUD) – only 53.3% of non-responders had a history of SUD (vs. 100%; *p* = 0.0075) and a lower number of psychiatric comorbidities among non-responders (*p* = 0.0381) (Table 2).

**Table 1:** Sociodemographic characteristics of treatment-resistant major depressive disorder subjects in short-term intravenous and oral ketamine intervention as stratified to responders and non-responders defined as a reduction of 50% or more in the Montgomery-Åsberg depression rating scale (MADRS) score between baseline and 7th infusion

Characteristic	All *N* = 40	Non-responders^1^*N* = 30	Responders^2^*N* = 10	*p*-value	Test statistic
Age in years (mean, 95% CI)	47.075 (42.429–51.721)	49.067 (43.652–54.481)	41.1 (31.4–50.8)	0.133^b^	t = 1.581_df = 16_
BMI (mean, 95% CI)	27.516 (25.841–29.191)	27.931 (26.043–29.819)	26.272 (22.084–30.46)	0.4363^b^	t = 0.802_df = 14_
Sex N (% of total N)					
Female	19/40 [47.5%]	14/30 [46.67%]	5/10 [50%]	1	
Male	21/40 [52.5%]	16/30 [53.33%]	5/10 [50%]		
Education N (% of total N)					
Elementary	2/39 [5.13%]	0/30 [0%]	2/9 [22.22%]	0.1152	
Vocational	4/39 [10.26%]	3/30 [10%]	1/9 [11.11%]		
Secondary	14/39 [35.9%]	12/30 [40%]	2/9 [22.22%]		
Higher	19/39 [48.72%]	15/30 [50%]	4/9 [44.44%]		
Unknown	1	0	1		
Employment status N (% of total N)					
Unemployed	7/40 [17.5%]	5/30 [16.67%]	2/10 [20%]	0.2036	
Pensioner^3^	10/40 [25%]	10/30 [33.33%]	0/10 [0%]		
Retired^4^	6/40 [15%]	4/30 [13.33%]	2/10 [20%]		
Employed	14/40 [35%]	9/30 [30%]	5/10 [50%]		
Studying	3/40 [7.5%]	2/30 [6.67%]	1/10 [10%]		
Marital status N (% of total N)					
Single	8/40 [20%]	6/30 [20%]	2/10 [20%]	0.4873	
Nonformal relationship	2/40 [5%]	1/30 [3.33%]	1/10 [10%]		
Married	24/40 [60%]	19/30 [63.33%]	5/10 [50%]		
Divorced	4/40 [10%]	2/30 [6.67%]	2/10 [20%]		
Widowed	2/40 [5%]	2/30 [6.67%]	0/10 [0%]		

Abbreviations: 1: Patients^*^ diagnosed with major depressive disorder who exhibited less than 50% reduction in Montgomery–Åsberg Depression Rating Scale (MADRS) scores from baseline to the 7th infusion during observational registry for safety and tolerability; 2: Patients diagnosed with major depressive disorder who exhibited a 50% or greater reduction in Montgomery–Åsberg Depression Rating Scale (MADRS) scores from baseline to the 7th infusion during observational registry for safety and tolerability; 3: person who is under retirement age but it is not able to work and gets social benefits; 4: person with pension above retirement age; b: T-test; c: Mann-Whitney U test; CI: Confidence Interval

^*^ Inpatients admitted to the Department of Psychiatry, Medical University of Gdańsk (Poland) between 2019 and 2022 (NCT04226963), and from 2023 onward (NCT05565352)

**Table 2:** Clinical baseline characteristics of treatment-resistant major depressive disorder subjects in short-term intravenous and oral ketamine intervention as stratified to responders and non-responders defined as a reduction of 50% or more in the Montgomery-Åsberg depression rating scale (MADRS) score between baseline and 7th infusion

Characteristic	All *N* = 40	Non-responders^1^*N* = 30	Responders^2^*N* = 10	*p*-value	Test statistic
Age of onset in years (median, Q1-Q3)	38 (22.5–44.5)	40 (22–47)	32.5 (24.75–35.75)	0.2093^c^	U = 184.5 (N1 = 29, N2 = 10)
Unknown	1	1	0		
Number of major depression episodes (mean, CI 95%)	3.972 (2.874–5.071)	3.25 (2.47–4.03)	6.5 (2.099–10.901)	0.1275^b^	t=-1.711_df = 8_
Unknown	4	2	2		
Duration of current episode in weeks (median, Q1-Q3)	22 (10.5–48)	24 (10–48)	13 (12–48)	0.802^c^	U = 67.5 (N1 = 25, N2 = 5)
Unknown	10	5	5		
Psychiatric comorbidities N (% of total N)					
Personality disorders	10/40 [25%]	7/30 [23.33%]	3/10 [30%]	0.6893	
Anxiety disorders	28/40 [70%]	21/30 [70%]	7/10 [70%]	1	
**Substance use disorder**	**26/40 [65%]**	**16/30 [53.33%]**	**10/10 [100%]**	**0.0075**	
Hisotry of trauma	17/40 [42.5%]	10/30 [33.33%]	7/10 [70%]	0.0663	
**Number of psychiatric comorbidities (median**,** Q1-Q3)**	2 (1–3)	2 (1–2)	3 (2-3.75)	**0.0381** ^**c**^	**U = 85.5** (N1 = 30, N2 = 10)
Family history of psychiatric disorders N (% of total N)	20/40 [50%]	15/30 [50%]	5/10 [50%]	1	
Somatic comorbidities N (% of total N)					
Hypertension	13/40 [32.5%]	11/30 [36.67%]	2/10 [20%]	0.4507	
Diabetes mellitus	1/40 [2.5%]	1/30 [3.33%]	0/10 [0%]	1	
Hyperlipidemia	7/40 [17.5%]	7/30 [23.33%]	0/10 [0%]	0.1612	
Stroke	3/40 [7.5%]	3/30 [10%]	0/10 [0%]	0.5597	
Epilepsy	2/40 [5%]	1/30 [3.33%]	1/10 [10%]	0.4423	
Others	11/40 [27.5%]	9/30 [30%]	2/10 [20%]	0.6962	
Number of somatic comorbities (median, Q1-Q3)	1 (0-1.25)	1 (0–2)	0 (0–1)	0.0788^c^	U = 203.5 (N1 = 30, N2 = 10)
Suicidality N (% of total N)					
Prior	22/40 [55%]	18/30 [60%]	4/10 [40%]	0.3003	
Current	12/40 [30%]	8/30 [26.67%]	4/10 [40%]	0.4507	
Prior ECT N (% of total N)	6/40 [15%]	6/30 [20%]	0/10 [0%]	0.3074	
History of benzodiazepines use N (% of total N)	18/40 [45%]	15/30 [50%]	3/10 [30%]	0.4645	
Length of stay in hospital before (median, Q1-Q3)	11 (5-34.5)	11 (4-33.5)	18.5 (6-38.5)	0.361^c^	U = 112 (N1 = 28, N2 = 10)
Unknown	2	2	0		
MADRS total score					
At screening (mean, 95% CI)	28.15 (26.217–30.083)	28.9 (26.505–31.295)	25.9 (22.838–28.962)	0.107^b^	t = 1.676_df = 23_
**Before 7th infusion (mean**,** 95% CI)**	**18.525 (15.242–21.808)**	**22.967 (20.156–25.777)**	**5.2 (2.82–7.58)**	< 0.0001 *** ^**b**^	**t = 10.266**_**df = 35**_

Abbreviations: 1: Patients diagnosed with major depressive disorder who exhibited less than 50% reduction in Montgomery–Åsberg Depression Rating Scale (MADRS) scores from baseline to the 7th infusion during observational registry for safety and tolerability; ^2^ Patients diagnosed with major depressive disorder who exhibited a 50% or greater reduction in Montgomery–Åsberg Depression Rating Scale (MADRS) scores from baseline to the 7th infusion during observational registry for safety and tolerability; b: t-test; c: Mann-Whitney U test; CI: Confidence Interval

^*^ Inpatients admitted to the Department of Psychiatry, Medical University of Gdańsk (Poland) between 2019 and 2022 (NCT04226963), and from 2023 onward (NCT05565352)

## Discussion

This study’s results indicate that baseline SUD comorbidity and a higher number of psychiatric comorbidities are associated with more favourable treatment outcomes with ketamine as adjunctive treatment to the standard of care in TRD. No differences between psychometric baseline scores were observed.

The notably lower prevalence of SUD among non-responders is particularly striking, given the well-documented high burden of comorbidities in patients with TRD [5]. This finding may be partially explained by emerging evidence highlighting ketamine’s distinct effects on specific clinical domains [8, 9, 12]. For instance, ketamine has demonstrated significant efficacy across various SUD subtypes, including cocaine, opioid, and alcohol use disorders, by enhancing motivation for cessation, reducing cravings, and decreasing both the frequency and quantity of substance use [12, 13]. However, findings from the REAL-ESK study indicate that TRD patients with comorbid SUD exhibit treatment responses comparable to the broader trial population, reinforcing ketamine’s multifaceted and domain-specific therapeutic potential in TRD management [14, 15]. Furthermore, the observed disparity in SUD prevalence between responders and non-responders reflects an existing trend in literature, suggesting that comorbid SUD may potentiate, rather than hinder, ketamine’s antidepressant effects [16]. Mechanistically, ketamine’s capacity to modulate glutamatergic dysregulation and normalize activity in prefrontal and mesolimbic pathways central to SUD pathophysiology is pivotal [12, 17, 18]. Nonetheless, differences in the prevalence of SUD reported might also, at least in part, reflect a sampling bias in participants’ recruitment. Moreover, ketamine’s alleviating effects on anhedonia are likely attributable to its activation of the reward system, which may contribute to distortion of response assessment among patients burdened with SUD [9]. However, while patients with a history of SUD were eligible for recruitment, ongoing substance abuse was not permitted. The risk of ketamine misuse remains a topic of ongoing debate, particularly as rates of ketamine abuse have risen in recent years [19]. This trend is especially evident among adults with depressive symptoms, potentially reflecting attempts at self-medication [19, 20].

The lower number of psychiatric comorbidities represents a surprising finding, as currently, there is no other evidence or analysis on multiple psychiatric comorbidities as an impactful feature of ketamine response. However, several hypotheses might be generated to explain our findings. Firstly, patients with fewer psychiatric comorbidities may have TRD driven by “pure” MDD rather than additive comorbidities. Non-responders may represent a distinct TRD subtype, characterized by fewer comorbidities and potentially involving less understood and more ingrained neurobiological disruptions or neurodegenerative components [21]. Conditions such as anxiety or SUD, often accompanied by heightened affective dysregulation and behavioral distress, might amplify the perceived benefits of ketamine’s rapid-acting antidepressant effects. In contrast, patients without comorbidities may exhibit less apparent symptomatic improvement, despite potential underlying neurobiological changes. Evidence supporting the hypothesis that such neurobiological changes may not be immediately detectable in short-term interventions comes from the ESCAPE trial, which demonstrated significantly higher remission rates when the duration of S-ketamine treatment was extended from 8 weeks to 32 weeks (increasing from 27.7 to 49.1%) [22]. In the context of TRD downstaging hypotheses, non-responders may represent those at the most advanced stage of the disorder, while responders appear to be situated at a milder phase [23]. Consequently, the disease-modifying effects are more readily observable via psychometric scales in the lower stages of TRD compared to its more severe manifestations, even if the downstaging process in non-responders was achieved [23].

Based on psychometric assessments, baseline MADRS scores did not differ between responders and non-responders. These results are in line with the heterogeneity of existing literature. Chen et al. described baseline depression severity as one of “the strongest non-response predictors,” and Miller et al., conversely, as a “key factor of response.” while an analysis by Lucchese et al., no difference was detected at baseline depression severity [24–26].

The relatively low proportion of responders compared to published results might be attributed to multiple factors [27, 28]. In the study by Loo et al., ketamine was administered subcutaneously, which could result in lower bioavailability compared to the intravenous administration used in our study, making direct dosing comparisons challenging [27]. Notably, in their second cohort, where flexible dosing was allowed up to 0.9 mg/kg, higher remission and response rates were observed. Furthermore, in their second cohort with higher ketamine dosages, the response rate was 29%, which is comparable to our findings. Similarly, another study utilizing also higher intravenous ketamine doses, up to 1 mg/kg, also reported a response rate of approximately 29% (but higher when 0.75-1 mg/kg were used) [28].

The clinical profile of non-responders provides valuable insights into identifying patients who may benefit from alternative TRD interventions beyond ketamine. Non-responders demonstrated a lower prevalence of SUD and psychiatric multiple comorbidity burden. These features are related to neurobiological disruptions, which were effectively addressed by ketamine’s pharmacological mechanisms [12, 15, 29]. They are well-established risk factors for TRD, prompting the question of whether ketamine might introduce a paradigm shift by exerting a disease-modifying role in TRD [5]. If further research confirms ketamine’s efficacy in TRD patients burdened with SUD or multiple psychiatric comorbidities, it could be positioned as a targeted, disease-modifying treatment for these populations. This concept aligns with earlier proposals regarding ketamine’s potential to downstage TRD severity [23]. From this perspective, non-responders should be considered a distinct, more refractory subtype of TRD that requires therapeutic strategies utilizing mechanisms of action different from that of ketamine, e.g. electroconvulsive therapy (ECT). ECT is regarded as one of the most effective interventions for TRD [4]. However, recent RCT in TRD (*n* = 365) demonstrated the noninferiority of ketamine to ECT, challenging the paradigm of ECT as the only gold-standard intervention [30]. Moreover, ECT response predictors differ from ketamine non-responder characteristics, with older age, absence of medication resistance, shorter duration of the current depressive episode, and severe MDD with psychotic features associated with ECT response [31, 32]. Additionally, none of the patients exhibited psychotic features, which may partially explain no responders with prior ECT in the study’s population. While there were initial concerns that ketamine could worsen psychotic symptoms, current evidence suggests it is safe [33]. The lack of responders who underwent ECT may be, in part, attributed also to patient preferences and ECT availability. In the scope of psychometrics, no significant result in this study and heterogeneity of evidence in literature underscore the complex and often contradictory nature of predictors for ketamine response in TRD, highlighting the need for further investigation into how assessment methods may impact the interpretation of baseline characteristics.

## Strengths and limitations

Our findings hold particular relevance for three key reasons. First, this study uniquely examines a population of patients with TRD non-responding to short-term ketamine administration, offering critical insights into a demographic that remains underrepresented in clinical research. Second, by leveraging real-world evidence, our approach captures the intricacies of clinical practice, reflecting the multifaceted realities encountered in everyday care, a stark contrast to the highly controlled environments of randomized controlled trials. Lastly, treatment response was evaluated using the MADRS, a widely recognized gold standard among clinician-rated instruments for assessing depression severity, thereby ensuring methodological rigor and robustness in outcome measurement.

Nevertheless, several limitations of our findings are important to note. Firstly, this is the post-hoc study based on data from the naturalistic observational design trials. This condition makes it impossible to distinguish the role of nonspecific factors, including the placebo effect. Moreover, sample sizes are relatively small, which may concern issues associated with statistical power and a low number of statically significant results. Differences in ketamine’s route of administration and dosage also may present an impact on final results. However, a recent open-label RCT found no significant score differences between intravenous (0.5 mg/kg) and oral (150 mg) ketamine at days 14 and 30 [34]. Moreover, given that the bioavailability of oral ketamine is approximately 20%, the oral dosages used were four to five times higher than the intravenous dose to account for the differences in bioavailability [35]. As the observed intervention was an adjunctive treatment to currently used oral antidepressants, heterogeneity of basal pharmacology treatment might influence the measured effects. Additionally, the quality of data retrieved from EMRs is limited to the scrupulous work of clinicians. Finally, we lack detailed patient accounts of their treatment, which would help understand patient-centered and functional outcomes beyond those the limited data that are reflected on the MADRS questionnaire.

## Conclusions

This study provides key insights into the population of TRD non-responders treated with ketamine, including an exhibition of lower rates of SUD and a lower number of psychiatric comorbidities among them. It suggests a higher burden by traditional TRD risk factors might not limit ketamine efficacy and even facilitate response compared to MDD. Understanding this relationship is essential, as it plays a critical role in determining whether to initiate or continue treatment for TRD. Early identification of potential non-responders can significantly reduce exposure to ineffective ketamine interventions and facilitate timely transitions to alternative treatments. Clarifying the factors that influence ketamine response enhances the understanding of TRD management and lays the groundwork for future research aimed at optimizing therapeutic strategies.

## Data Availability

Data available from corresponding author upon reasonable request.

## Abbreviations

MDD: major depressive disorder
YLDs: years lived with disability
TRD: treatment-resistant depression
EMA: European Medicines Agency
FDA: Food and Drug Administration
RAADs: rapid-acting antidepressants
NMDAR: NMDA receptor
*iv*: intravenous
MADRS: Montgomery-Åsberg Depression Rating Scale
SUD: substance use disorder
